# SCO-spondin knockout mice exhibit small brain ventricles and mild spine deformation

**DOI:** 10.1186/s12987-023-00491-8

**Published:** 2023-12-05

**Authors:** Huixin Xu, Guillaume P. Dugué, Yasmine Cantaut-Belarif, François-Xavier Lejeune, Suhasini Gupta, Claire Wyart, Maria K. Lehtinen

**Affiliations:** 1 Department of Pathology, Boston Children’s Hospital and Harvard Medical School, Boston, MA 02115 USA; 2 Neurophysiology of Brain Circuits, Institut de Biologie de l’Ecole Normale Supérieure (IBENS), Ecole Normale Supérieure, CNRS, INSERM, Université PSL, 75005 Paris, France; 3 Sorbonne Université, Paris Brain Institute (Institut du Cerveau, ICM), Institut National de la Santé et de la Recherche Médicale (INSERM) U1127, Centre National de la Recherche Scientifique (CNRS) Unité Mixte de Recherche 7225, Assistance Publique–Hôpitaux de Paris (APHP), Campus Hospitalier Pitié-Salpêtrière, 47, bld Hospital, 75013 Paris, France

**Keywords:** SCO-spondin/SSPO, Subcommissural organ (SCO), Reissner’s fiber, Cerebrospinal fluid, Spinal cord, Ventricular volume, Spine deformities

## Abstract

Reissner’s fiber (RF) is an extracellular polymer comprising the large monomeric protein SCO-spondin (SSPO) secreted by the subcommissural organ (SCO) that extends through cerebrospinal fluid (CSF)-filled ventricles into the central canal of the spinal cord. In zebrafish, RF and CSF-contacting neurons (CSF-cNs) form an axial sensory system that detects spinal curvature, instructs morphogenesis of the body axis, and enables proper alignment of the spine. In mammalian models, RF has been implicated in CSF circulation. However, challenges in manipulating *Sspo*, an exceptionally large gene of 15,719 nucleotides, with traditional approaches has limited progress. Here, we generated a *Sspo* knockout mouse model using CRISPR/Cas9-mediated genome-editing. *Sspo* knockout mice lacked RF-positive material in the SCO and fibrillar condensates in the brain ventricles. Remarkably, *Sspo* knockout brain ventricle sizes were reduced compared to littermate controls. Minor defects in thoracic spine curvature were detected in *Sspo* knockouts, which did not alter basic motor behaviors tested. Altogether, our work in mouse demonstrates that SSPO and RF regulate ventricle size during development but only moderately impact spine geometry.

**Supplementary Information:**

The online version contains supplementary material available at 10.1186/s12987-023-00491-8.

## Introduction

The subcommissural organ (SCO) is a circumventricular organ that shares some functions with the choroid plexus (ChP), the principal source of cerebrospinal fluid (CSF) and a blood-CSF barrier. Like the ChP, the SCO secretes a range of regulatory proteins into the CSF including growth factors and binding proteins (e.g., Transthyretin) that may contribute to CNS development [1]. The SCO is also hypothesized to regulate ventricular volume like the ChP [2], but our understanding of SCO functions is very limited.

In adult organisms the SCO is the sole source of a unique member of the spondin family of proteins termed SCO-spondin (SSPO), a gigantic glycoprotein (close to 5000 amino acids, see [3]). Soluble monomeric SSPO can fold in different configurations and its numerous motifs enable interactions with diverse signaling molecules in the CSF including monoamines, growth factors, and lipids [4, 5]. Like other so-called matricellular proteins, SSPO is likely to have a diversity of functions mirroring its structural complexity. In addition to maintenance of CSF flow dynamics, proposed functions of SSPO include neurogenesis and neuroprotection [6–9]. SSPO may also serve to regulate bioavailability of substances in the CSF when its binding capacity is sufficient to limit the concentration of unbound ligands. In addition to its soluble monomeric form, SSPO can polymerize to form Reissner’s fiber (RF), a long extracellular threadlike protein aggregate that emerges during embryogenesis and runs from the SCO through the cerebral aqueduct, the fourth ventricle, and into central canal of the spinal cord [10]. Despite its presence across numerous vertebrate species, the function of the RF in mammals is unclear. However, there is good evidence that the RF plays a critical role in developing zebrafish [11]. Together with CSF-contacting neurons (CSF-cNs), the RF forms an axial sensory system that has been shown in zebrafish to detect spinal curvature, instruct morphogenesis of the body axis, and enable proper alignment of the spine [12–14].

Here, we leverage a *Sspo* knockout (*Sspo*^−/−^) mouse that we generated using CRISPR/Cas9-mediated genome editing to explore the functions of SSPO in mice by determining the effects of loss of SSPO on ventricular volume, spine curvature and motor function. We found that the brain ventricle volumes were reduced in *Sspo*^−/−^ mice compared to their wild type (*Sspo*^+/+^) siblings. Unlike zebrafish, we observed that *Sspo*^−/−^ mice had only minor defects in spine geometry that were confined to the thoracic level and had no performance deficits in basic motor assays. Altogether, our work demonstrates in mice that SSPO and RF are involved in regulating CSF volume during development but probably play a minor role in regulating spine geometry.

## Methods

### Animals and genome editing

All experiments involving mice in this study were approved by the BCH IACUC. Germline *Sspo* knockout (*Sspo*^*tm1Leh*^, referred to in the Results and Discussion as *Sspo*^*−/−*^) mice were generated by genome editing on a C57BL/6 background. Animals were housed in a temperature-controlled room on a 12-h light/12-h dark cycle and had free access to food and water. A comparable number of males and females were included. *Sspo*^+/+^ and *Sspo*^−/−^ mice were littermates generated by breeding heterozygous males and females.

*Sspo*^*tm1Leh*^ mice were generated using the following guide RNA:

Sspo-Ex2-1 TCCATAGCATCCCAAAGAGA

Sspo-Ex3-1 GGTACGAGGTCCTCCTGACG

Sspo-Ex4-1 GTAGCAGGCCTGGTTTCCGG

Sspo-Ex6-1 CAGCTAGCAAGTAGGTACAC

Progeny were screened for loss of restriction enzyme sites for successful modification and sequenced to determine the consequences of genetic modifications. Of all the mice screened, one founder carried a 5 bp deletion in exon 2, leading to an early stop codon after 11 amino acids. The founder was used to establish the *Sspo*^*tm1Leh*^ colony. Subsequent genotyping was conducted using the following primer set: CTGTGAAGGGATGCTGGAGGTAG (forward) and GTACTATGAATCCGAGGCCCCAA (reverse). The product from wild type mice is a 426 bp band that can be cut by HpyAV. Mice carrying the 5 bp deletion will yield a band that cannot be digested. Later genotyping was conducted at Transnetyx.

### Immunohistochemistry

**Fig. 1:**
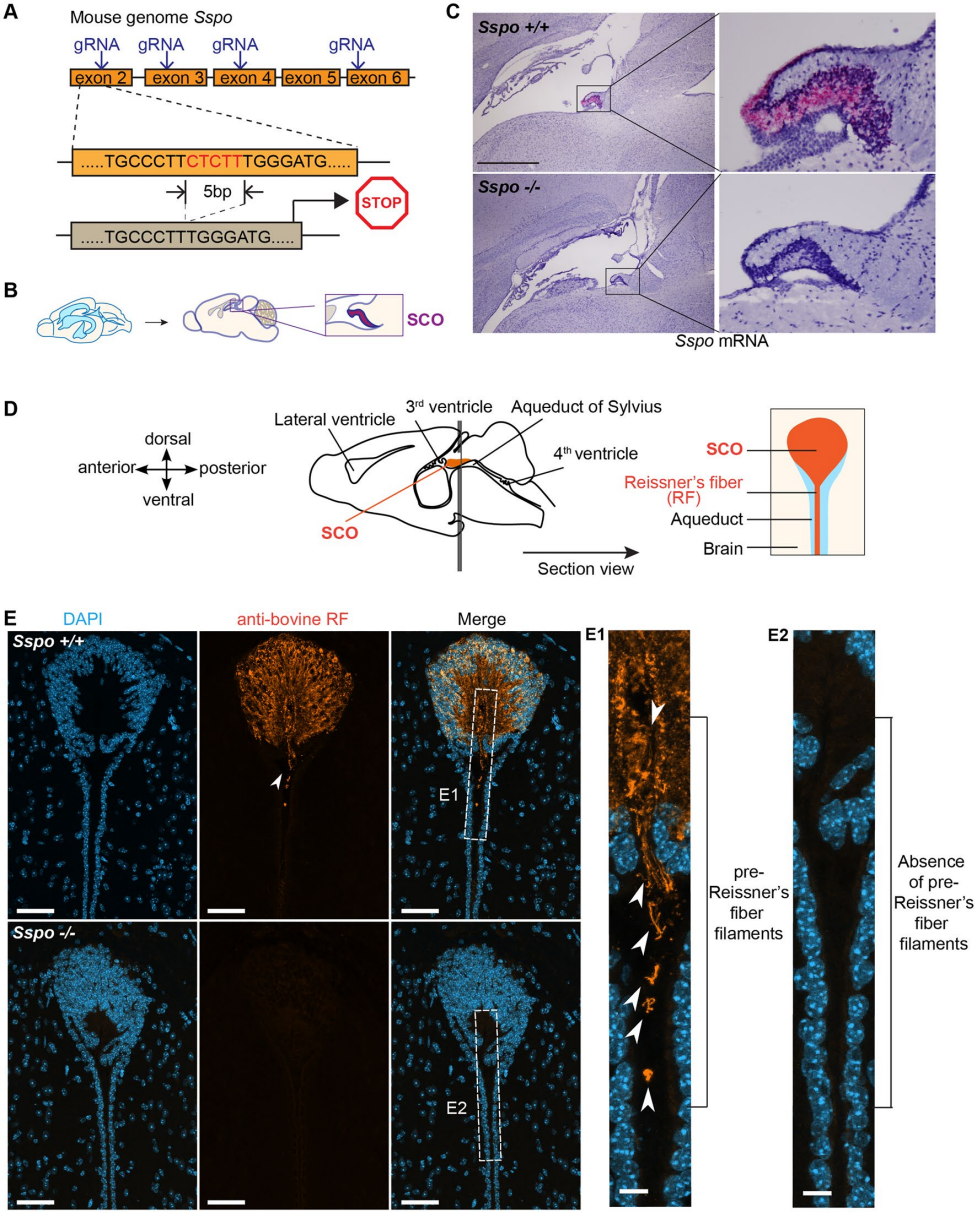
*Sspo*^−/−^ mice show a normal SCO but lack RF positive material in the SCO and ventricles. **A** Schematic depicting gene editing strategy. **B** Schematic depicting SCO location in sagittal brain sections corresponding to **C**. **C** BaseScope reveals lack of *Sspo* transcripts in the SCO of *Sspo*^−/−^ mice (12 weeks). Scale = 1 mm (left panels) and 200 µm (right panels). **D** Schematic depicting the brain sectioning strategy taken to visualize SCO ependyma and proximal ventricular region. **E** RF positive material was not detected in *Sspo*^−/−^ mice vs. controls. Scale = 50 µm

Mice were perfused intracardially with ice-cold PBS followed by cold 4% PFA. Brains were dissected, soaked in 4% PFA at 4 °C overnight and cryopreserved in 30% sucrose for 2 days before snap-freezing in liquid nitrogen-cooled isopentane. Coronally oriented cryosections were prepared at 16 µm thickness using a CM3050S cryostat (Leica). Blocking was performed by incubating the sections in 3% BSA and 0.1% Tween-20 in PBS one hour at room temperature. Sections were then incubated overnight at 4 °C with the L1P1b rabbit anti-serum (kind gift of S. Gobron; 1:250 dilution) [15] to detect RF-positive material in the SCO and ventricles. The L1P1b anti-serum was generated by immunizing rabbits with freshly dissected bovine RF and was previously shown to react exclusively with the secretion products of the SCO [15]. Therefore, it is specific to “all” RF-material including the SCO-spondin protein. After washing with 0.1% Tween-20 in PBS, sections were incubated with a goat-anti-rabbit-Alexa488 secondary antibody (Invitrogen; 1:400 dilution) for 45 min at room temperature with counterstaining by 4ʹ,6-diamino-2-phenylindole (DAPI) to label nuclei. Sections were imaged on an inverted SP8 DLS confocal microscope (Leica) equipped with a 40X objective (oil immersion; NA = 1.3). The same imaging parameters (laser power, scanning speed, wavelength gating for photon collection) were used to image sections derived from wild type and knockout animals. Images were processed using Fiji [16]. Maximal Z-projections of 10 µm in depth are represented (Fig. 1).

### BaseScope

Mouse brain sections were prepared as described above in RNAse-free conditions and stored at − 80 °C. BaseScope (ACD Bio, 323910) was used to detect the presence or absence of *Sspo* mRNA. A customized probe (1zz probe named BA-Mm-sspo-1zz-st, targeting 45–85 of NM_173428.4 *Sspo* sequence) was designed to distinguish wild type sequence vs. mutant sequence with 5 bp deletion. Hematoxylin staining was applied to visualize the rest of the tissue.

### Magnetic resonance imaging (MRI)

Mice were imaged using Bruker BioSpec small animal MRI (7T) at 6 weeks while under anesthesia by isoflurane. A warm pad was used to maintain body temperature. Breathing rate and heart rate were monitored to reflect the depth of anesthesia. All axial T2 images were acquired using the following criteria: TE/TR = 60/4000; Ave = 8; RARE = 4; slice thickness = 0.6 mm.

### Calculation of the ventricular volume

**Fig. 2:**
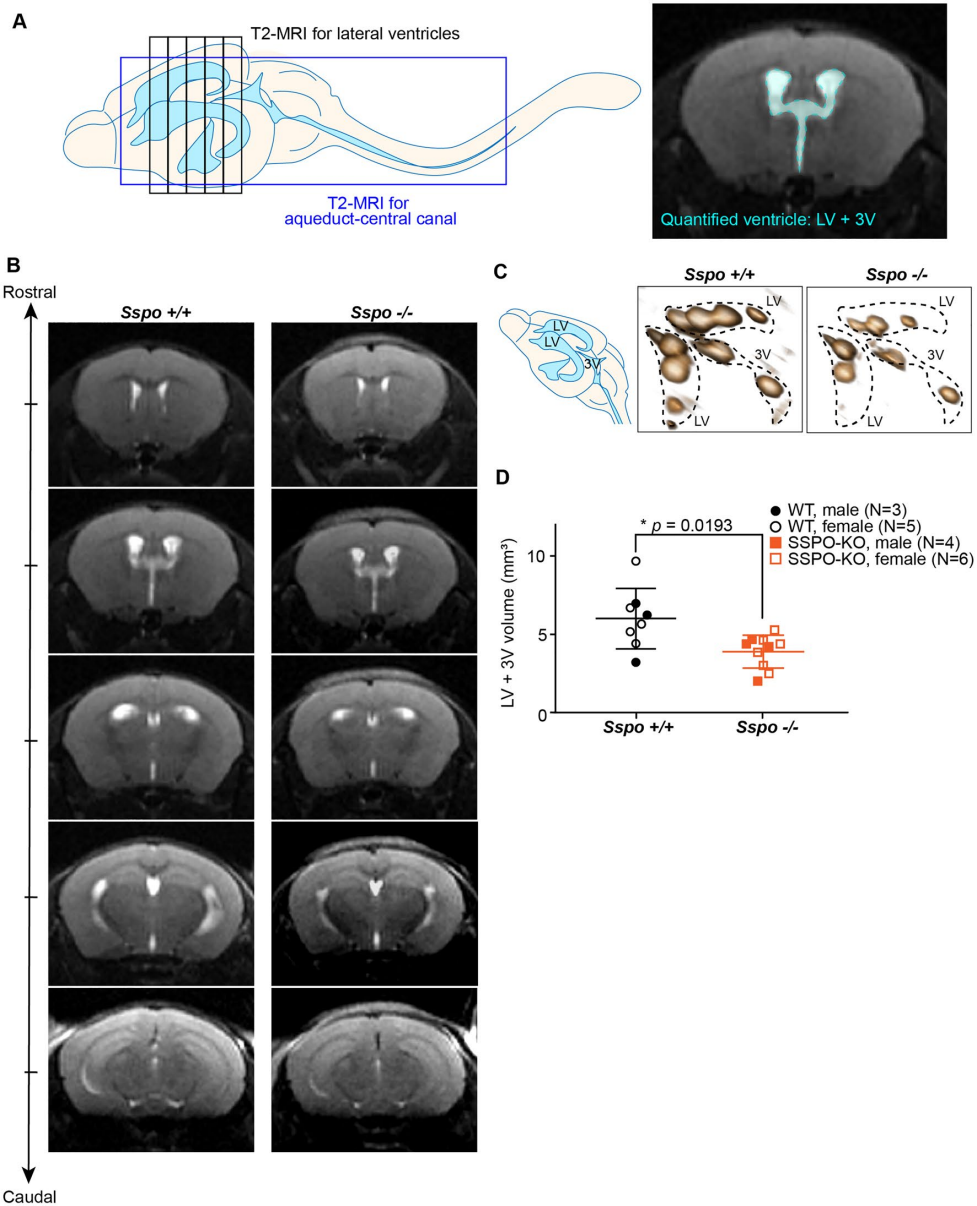
*Sspo*-deficiency leads to reduced ventricle volume at 6 weeks. **A** Schematic depicting brain regions captured by MRI and manual ventricle segmentation. **B** Representative sequential MR images of *Sspo*^+/+^ and *Sspo*^−/−^ brains, showing reduced ventricle (white) sizes in *Sspo*^−/−^ mice. **C** 3D rendering of lateral and third ventricles from *Sspo*^+/+^ and *Sspo*^−/−^ mice. **D** Quantification of lateral and third ventricle volumes. Male and female mice were analyzed and plotted together but marked separately. * *p* = 0.0193, Welch’s t-test. Data presented as mean ± S.D

Ventricle volumes were calculated by manual segmentation using FIJI/ImageJ. Lateral ventricle and 3rd ventricle areas, which appear white in T2 MR images, were measured by manual quantification of the white patches (determined by greyscale) in FIJI, similar to approaches used in previous studies [17–19]. All selections were blinded to avoid selection bias. Ventricle CSF volume was calculated by total ventricle area × 0.6 mm slice thickness. To avoid bias, all images were identified based on mouse date of birth and tag ID without information about genotypes. Calculation was completed for each mouse without knowledge of their genotypes. After all data were documented, a separate file containing mouse ID and genotype information was used to assign genotype to each datapoint—see also [19]. Representative 3D images in Fig. 2C were created by Paraview with automated segmentation to aid with visualization of ventricular structure. 2D MRI images without visible ventricles resulted in an empty space in the 3D reconstruction. The images were not used for quantitative analysis.

### Behavioral analysis

All behavioral tests were conducted by staff in the BCH Animal Behavior and Physiology Core who were blinded to the mouse genotypes.

#### Gripping test

We used a BIO-GS4 system to quantify the forelimb gripping strengths of mice. This test determines the maximal peak force generated by each mouse as the operator removes it from a specially designed grid.

#### Rotarod

Rotarod was performed using the IITC Rotarod system. Mice were placed on a rotating beam (1¼ inches diameter) with rotation frequency increasing from 5 r.p.m. to 40 r.p.m. over the course of 5 min. The length of time (seconds) each mouse stayed on the beam was recorded. Each mouse was tested in 6 independent trials.

#### Digigait

Mice were placed on a Digigait device which resembles a treadmill. A series of parameters describing the way the mice step forward was recorded, including time spent at swing, brake, propel, stride, and stance; length and frequency of strides; paw angle and its variability; and stance width. Each paw was analyzed separately.

#### Open field

Tests were conducted using Open Field Systems by Kinder Scientific. The mice were placed inside an open space with high density infra-red beams. The movements of the mouse were recorded automatically based on its crossing of the infra-red beams. Total travel distance, rearing frequency, and time spent at center vs. corners of the field were analyzed over the course of 30 min.

### Micro-CT imaging and analysis of spine morphology

Micro-computed tomography (micro-CT) skeletal scans were acquired using an Albira system (Bruker). Animals were lightly anesthetized by 3% isoflurane and placed in a prone position on the micro-CT bed. Special attention was paid to ensure that the spine was not artificially bent, and that all limbs were in a relaxed position and not pinned underneath the torso. Stacks of cross-sectional images were reconstructed with a volume resolution of 125 μm.

**Fig. 3:**
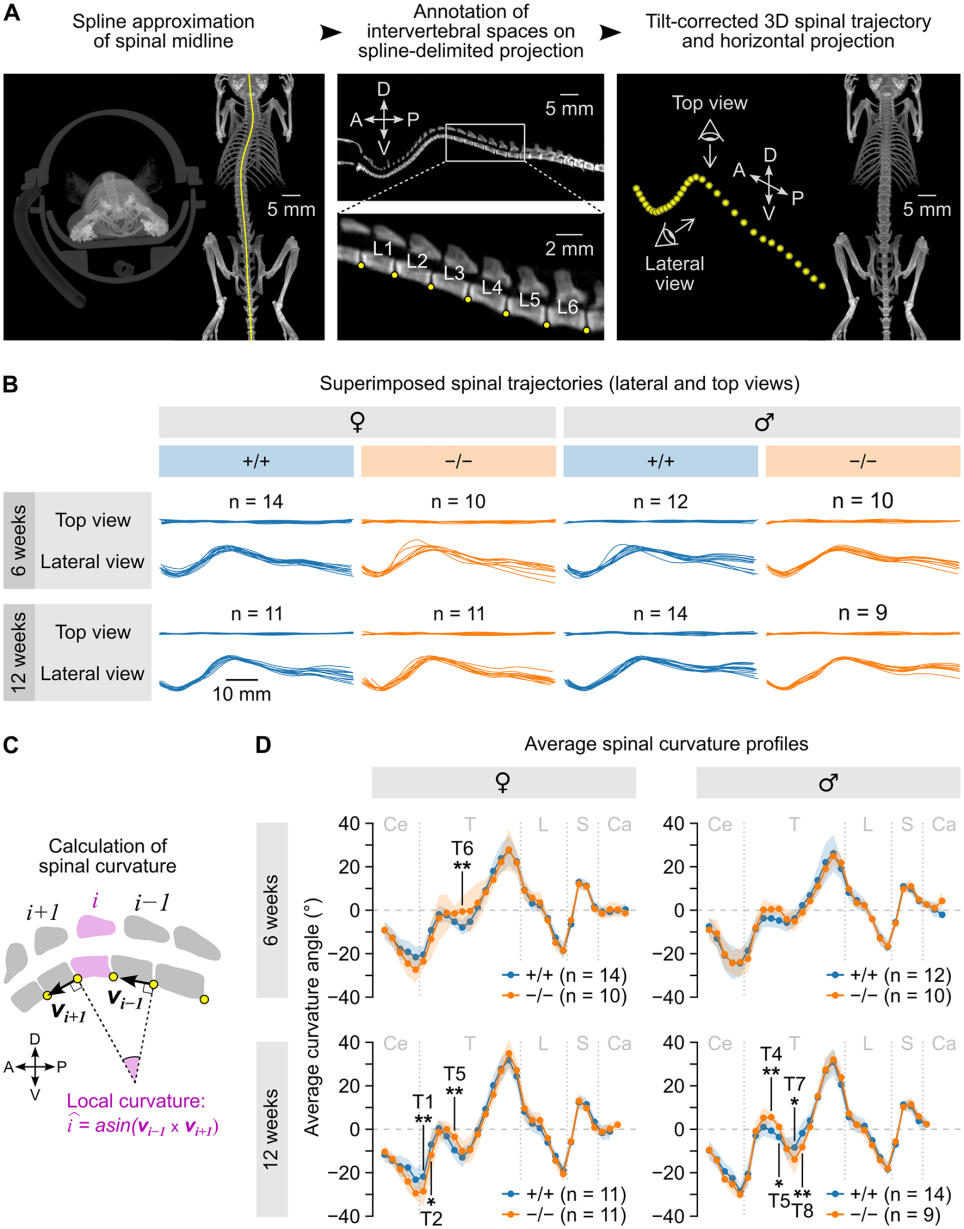
*Sspo*-deficiency has a mild impact on spinal curvature in the thoracic level. **A** Principle of the biplanar annotation approach used to measure three-dimensional spinal trajectories. A spline approximation of the spinal midline was first obtained on a horizontal projection image (right image in left panel); reference points (yellow dots) were then placed at the ventral aspect of intervertebral spaces on a lateral projection image computed from a stripe of voxels along the spline (middle); their medio-lateral position was interpolated using the spline, yielding a set of Cartesian coordinates describing the three-dimensional trajectory of the spine (right). The example mouse shown here was slightly tilted to the left, as shown by the coronal projection (left image in the left panel), resulting in an apparent deviation of the spine in the horizontal projection (right image in left panel); this apparent deviation disappeared after applying the corrective rotation computed using the three-dimensional trajectory (horizontal projection in right panel). A: anterior; D: dorsal; P: posterior; V: ventral. **B** Optimal alignments of individual spinal trajectories for wild type (*Sspo*^+/+^) and *Sspo*^−/−^ mice grouped by gender and age (6 and 12 weeks). In each case, top and lateral views are shown (top and bottom curves, respectively). **C** Sketch detailing the calculation of local curvature angles. **D** Average curvature angle (± S.D., shaded area) at each vertebral position computed across mice grouped by gender, age (6 and 12 weeks) and genotype. Vertebral levels at which significant differences between *Sspo*^−/−^ and *Sspo*^+/+^ mice were found using linear mixed-effects models followed by post hoc pairwise comparisons are indicated (*: *p* < 0.05; **: p < 0.001; see values in the text). Vertical gray dashed lines indicate regional limits (Ce: cervical; T: thoracic; L: lumbar; S: sacral; Ca: caudal)

A custom pipeline written in Python (https://github.com/teamnbc/MouSpine) was designed to compute three-dimensional spinal trajectories using stacks of micro-CT cross sectional images. The pipeline implements a series of annotation steps as follows. A horizontal maximum intensity projection (MIP) image is first used to place a set of points along the spinal midline. A function connecting these points is computed by cubic spline interpolation and used as an approximation of the midline trajectory (Fig. 3A, left). In this image, the user can also register the antero-posterior position of one reference vertebra (e.g., L6). A lateral MIP is then computed using a thin vertical stripe of voxels centered around the spinal midline, yielding an image in which the spinal canal is clearly visible from head to tail. The user can then annotate a series of vertebral landmarks (reference points) in this image. The ventral limit of intervertebral spaces appeared as robust landmarks and were used as reference points in this study (Fig. 3A, middle). Using the known position of the reference vertebra defined previously, the pipeline automatically associates these points to specific vertebral identities. In the present study, each reference point was associated with the vertebra immediately anterior to it (e.g., the L6 point was located at the posterior side of L6). At this stage, only the antero-posterior and dorso-ventral coordinates of reference points are defined. The final step consists in interpolating their medio-lateral coordinates using the spline function calculated above and describing the spinal midline, yielding a set of Cartesian coordinates describing the three-dimensional trajectory of the spine (Fig. 3A, right). For one mouse, the whole annotation process is typically completed in less than 2 min. The person who performed these annotations was blinded to the genotype of the mice.

The rest of the analysis was performed with custom code written in R (https://github.com/teamnbc/MouSpineR, using version 4.3.1), employing a right-handed reference frame in which the *x* axis points towards the nose, the *y* axis towards the left, and the *z* axis towards the dorsal side. The first step consisted of correcting for minor body misalignments (small lateral tilt and/or slightly slantwise body axis), which are inevitable when placing an animal on the micro-CT bed. For that purpose, spinal trajectories were centered and the roll and yaw rotations minimizing the sum of squared *y* coordinates were computed and applied. A few iterations of this process suppressed apparent lateral deviations visible on horizontal projection images in tilted animals, which were in fact the signature of their natural thoracic kyphosis seen at a slight angle (Fig. 3A, right). Individual spinal trajectories were then aligned using a second iterative process: in each iteration, the average position of reference points was calculated; then for each trajectory, the translation within the sagittal plane minimizing the sum of squared Euclidean distances of reference points relative to their average position was computed using a grid search algorithm and applied. This process rapidly converged (with less than 10 iterations) towards an optimal alignment of spinal trajectories. Spine curvature was computed as follows. Each vertebra *i* is flanked by a pair of reference points whose coordinates can be used to compute a three-dimensional unitary vector ***v***_***i***_ describing the orientation of its longitudinal axis. The local curvature around each vertebra *i* was calculated as the angle between the longitudinal axes of vertebrae *i − 1* and *i* + *1*, i.e. as the arcsine of the cross product ***v***_***i−1***_ × ***v***_***i+1***_ (Fig. 3C). Curvature profiles were obtained by plotting the resulting curvature angles as a function of vertebral identity, with positive and negative values representing convexities and concavities, respectively.

### Statistics

**Fig. 4:**
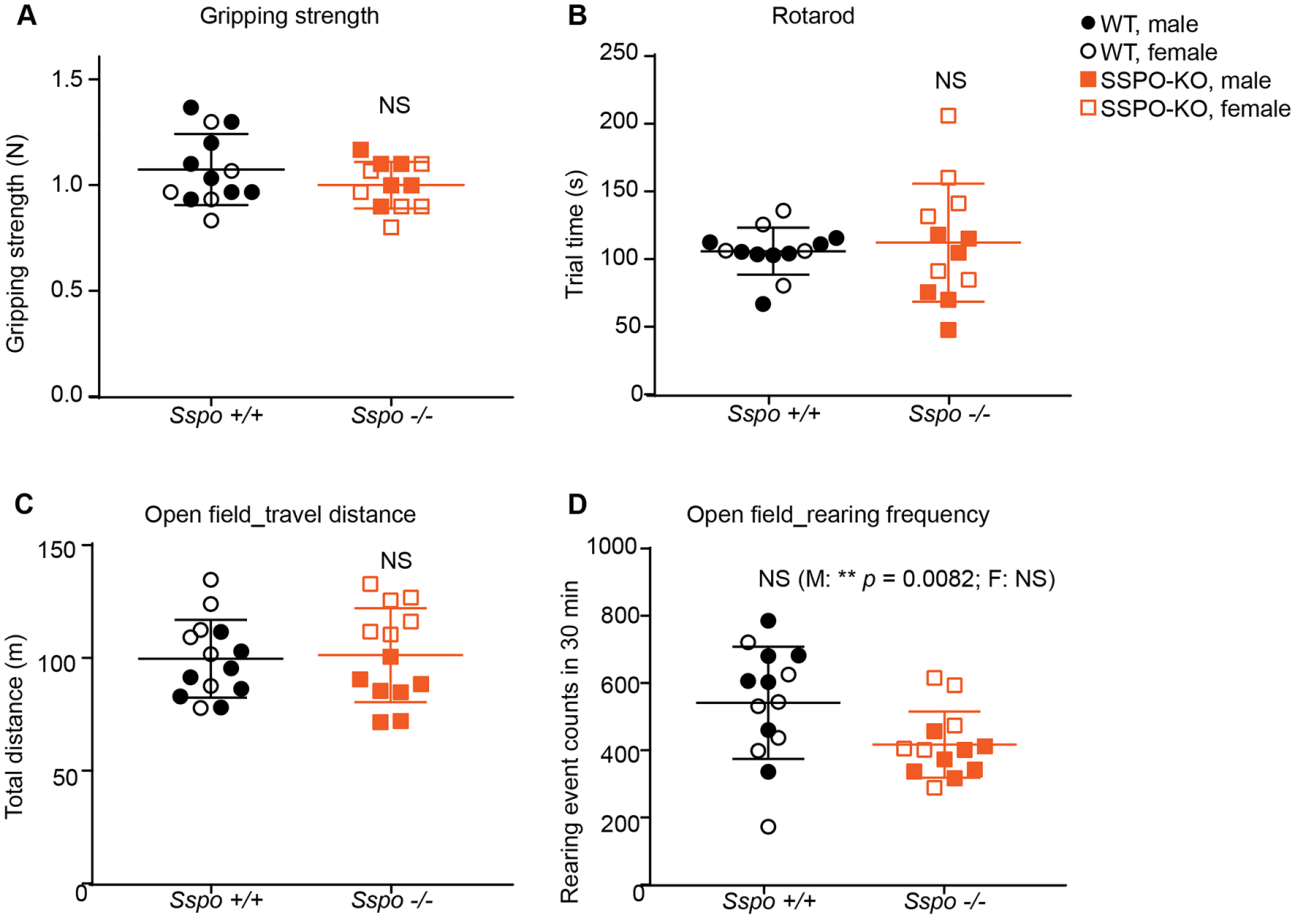
*Sspo*-deficiency does not alter basic motor tasks in 12–14 week old adult mice. No behavioral differences were observed in *Sspo*^−/−^ mice compared to wild type littermate controls in **A** forelimb gripping strength (not significant, N = 13 wild type, 8 males and 5 females, N = 12 *Sspo*^−/−^, 6 males and 6 females, *p* = 0.20), **B** average time spent on the rotarod (not significant, N = 13 wild type, 8 males and 5 females, N = 12 *Sspo*^−/−^, 6 males and 6 females, *p* = 0.64), **C** total distance traveled during each 30 min (not significant, N = 14 wild type, 7 males and 7 females, N = 13 *Sspo*^−/−^, 7 males and 6 females, *p* = 0.83). open-field test, and **D** number of rearing events during each 30 min (N = 14 wild type, 7 males and 7 females, N = 13 *Sspo*^−/−^, 7 males and 6 females, *p* = 0.0262 (significant *p* with Sidak correction for multiple comparison in open field test is 0.0170). If analyzing males only, ** *p* = 0.0082). Open field test. All panels have male and female marked separately and analyzed together unless specified. Welch’s t-test

All quantitative values are presented as mean ± standard deviation (S.D.) unless otherwise specified. Two-tailed t-tests were used for data presented in Figs. 2 and 4. Sidak correction for multiple comparison was used for Fig. 4D. For Fig. 3, all statistical analyses were conducted using R version 4.2.2 (R Development Core Team, 2022, https://www.r-project.org/). Linear mixed-effects model (LMM) analyses were performed for data presented in Fig. 3D. Group differences were examined using LMMs fitted to the curvature angles. In the fitted models, the factors genotype (*Sspo*^−/−^ vs *Sspo*^+/+^), region (thoracic, lumbar, or sacral), and sex (male vs. female), and their interaction terms were regarded as fixed effects. Cervical and caudal regions were excluded from the analysis as they were partially and variably covered by micro-CT scans depending on the positioning of the animal along the rostro-caudal axis. The mice identifier was assigned as a random (intercept) effect to account for the paired measurements by animal across the vertebral segments. Two separate LMMs were fitted, one for values from 6-week-old mice and one for values from 12-week-old mice, using restricted maximum-likelihood estimation (REML) with the function lmer in the lme4 package (v1.1-31) [20]. For each model, significance of the main effects and interaction terms was assessed based on Type II Wald chi-square tests using the function Anova in the car package (v3.1-1), followed by post hoc comparisons of the two genotypes at each vertebra within each sex using the emmeans package (v1.4.5) with False Discovery Rate (FDR) correction of p-values. The level of statistical significance was set at *p* or adjusted *p* < 0.05 for all tests.

## Results

### Reissner’s fiber was not detected in *Sspo* knockout mice

To study the function of RF during mammalian development, we generated an *Sspo* knockout mouse with the goal of eliminating its protein product, which aggregates to form the RF. Taking a genome editing approach, four guide RNAs were designed to target exons 2, 3, 4, and 6 of *Sspo*. All founders were screened for genetic modifications in all four regions targeted by gRNA. Of all the mutations identified in first generation progeny, one harbored a 5 bp deletion in exon 2 that shifted the open reading frame, resulting in an early stop codon after 11 amino acids (Fig. 1A). This founder did not harbor any additional genetic modifications. We established the *Sspo*^*tm1Leh*^ mouse strain from this founder, and we refer to it as *Sspo*^−/−^. *Sspo*^*−/−*^ mice were fertile and their body weight and size were not significantly different from their wild-type *Sspo*^+*/*+^ littermates. We kept the mice up to 12 months of age and did not observe any overt physical differences when compared to *Sspo*^+*/*+^ mice.

We validated loss of *Sspo* mRNA expression in the SCO of *Sspo*^−/−^ mice using BaseScope technology. BaseScope enables the sensitive detection of very small insertions or deletions of nucleotides in genomic DNA. BaseScope probes were designed to recognize the 5 bp deletion and to distinguish between wild type (*Sspo*^+/+^, successful binding) vs. KO (*Sspo*^−/−^, failed binding) samples. Brain sections from wild type littermates showed strong *Sspo* signal in the SCO region, while no signal was detected in the SCO region or ventricles of *Sspo*^−/−^ mice (Fig. 1B–C). The SCO structure and ependymal cells appeared unaffected by the genotypes (Fig. 1C, Additional file 3: Figure S1), and no obvious differences in cell polarity or nuclear arrangement were observed within the SCO. The absence of immunoreactivity against RF material in the SCO of *Sspo*^−/−^ mice (Fig. 1D, E) further confirmed the lack of functional *Sspo* transcripts. Importantly, while fibrous RF-positive structures were detected in wild type tissue, corresponding to loosely arranged bundles of thin filaments referred to as pre-RF material [21, 22], none were detected in *Sspo*^−/−^ mice (Fig. 1E, far right panels), indicating that no RF forms.

### *Sspo*-deficiency reduced brain ventricle volumes

We compared T2 weighted magnetic resonance imaging (MRI) brain scans of 6-week-old live *Sspo*^+/+^ and *Sspo*^−/−^ mice. MR images were segmented manually by researchers blinded to the animal IDs to calculate lateral ventricle volumes (Fig. 2A). We analyzed a total of 8 wild type *Sspo*^+/+^ mice (3 males and 5 females) and 10 *Sspo*^−/−^ mice (4 males and 6 females). We found that *Sspo*^−/−^ mice had on average a ~ 35% reduction in lateral and third ventricle size than *Sspo*^+/+^ littermate controls (Fig. 2B–D). These data were consistent across male and female mice and suggest that the absence of SSPO expression and RF formation during development limited lateral ventricle expansion. The overall brain sizes were also not significantly different as measured by the average brain area obtained from cross sections of MR images (*Sspo*^+/+^: 41.71 ± 2.44 mm^2^ vs. *Sspo*^−/−^: 43.13 ± 4.24 mm^2^, *p* = 0.4146, Welch’s t-test), suggesting the smaller ventricles were not due to a smaller brain phenotype. Additionally, we did not observe noticeable deficits in the ependyma surrounding the ventricles, white matter, the opening of the aqueduct, or the choroid plexus of *Sspo*^−/−^ mice (Additional file 3: Figure S2).

### *Sspo*-deficiency caused minor defects in the spinal geometry

To test if *Sspo*^−/−^ leads to abnormal spine morphology in mice as described in zebrafish [11, 23], we acquired whole-body micro-CT scans from *Sspo*^−/−^ mice and their *Sspo*^+/+^ littermates at 6 and 12 weeks of age. Because these scans did not reveal overt spine deformation, we developed a custom morphometric analysis pipeline (see “Methods”) to precisely estimate spinal geometry (Fig. 3A). By mapping the ventral aspect of intervertebral spaces along the spinal midline (hereafter designated as “reference points”), we obtained an accurate description of three-dimensional spinal trajectories. After correcting for minor misalignments related to body placement in the micro-CT tube (Fig. 3A), optimal alignments of spinal trajectories were computed and mean spinal trajectories were obtained by calculating the average position of reference points. Spinal trajectories clearly varied with age and gender but did not appear to differ notably between genotypes (Fig. 3B, Additional file 3: Figure S3A). A quantitative comparison of spinal geometries was performed by computing spinal curvature at each vertebral level (see “Methods” and Fig. 3C). The influence of three factors (genotype, spinal region and gender) on curvature angles was assessed independently for 6- and 12-week-old animals using linear mixed-effects models (see “Methods”). As expected from average spinal trajectories (Additional file 3: Figure S3), we found a significant interaction effect of spinal region and gender at both ages (*p* = 0.030 at 6 weeks and *p* < 2.2e−16 at 12 weeks, Type II Wald chi-square test). At 6 weeks, the model taking into account all three factors only showed a non-significant trend for a three-way interaction (p = 0.063, Type II Wald chi-square test) with a post hoc comparison indicating a difference in genotypes at the level of T6 in females (*p* = 0.004, see Additional file 1—detailed statistical report, Fig. 3D). At 12 weeks, a significant interaction effect of all three factors was found (*p* = 0.002, Type II Wald chi-square test) and the post hoc comparison of genotypes revealed differences at the levels of T1, T2 and T5 in females (*p* = 0.003, 0.043 and 0.007, respectively, Fig. 3D) and at the levels of T4, T5, T7 and T8 in males (*p* = 0.004, 0.048, 0.013 and 0.005, respectively, Fig. 3D). These results suggest that *Sspo*^−/−^ mice tend to build up abnormal spine curvature with age in the anterior thoracic region (T1–T8). Quantitatively, this effect can be considered as very mild since differences between average curvature angles of *Sspo*^−/−^ vs *Sspo*^+/+^ mice at these levels did not exceed 7.3 degrees, in contrast with established mouse models of scoliosis which exhibit changes in curvature of more than 10 degrees [24, 25].

### Sspo-deficiency did not impact basic motor behavior

*Sspo*-deficient mice retained many motor behaviors similar to controls. Male and female mice that were 12–14 weeks old were subjected to behavioral testing to determine if they showed any changes in muscle function and mobility. The mice were tested for gripping strength, balancing ability on rotarod, stride specifics by Digigait, and rearing and mobility in the open field. Overall, the *Sspo*^−/−^ mice lacked robust deficits in motor behaviors, except for a mildly reduced rearing frequency in males (Fig. 4 and Additional file 2). The lack of overt behavioral and mobility deficits in *Sspo*^−/−^ mice is consistent with their very mild spine deformation compared to controls.

## Discussion

Here, we report a new genetic mouse *Sspo*^−/−^ model created by CRISPR editing. Unlike Sspo deficient zebrafish [11, 23], *Sspo*^−/−^ mice exhibited very mild spine deformities confined to the thoracic level and no major motor phenotype. These findings likely reflect species-specific mechanisms regulating spine geometry. Intriguingly, studies in chicken embryos suggest that when SSPO is first secreted in its soluble form by the SCO into the CSF, it has neurogenic properties and contributes to neurogenesis [26]. We did not observe any evidence of microcephaly or other overt changes in mouse brain.

Our data also diverge from previous reports that SCO damage or malformation and lack of RF in mice and rats caused spontaneous hydrocephalus with aqueductal stenosis [27–34]. Instead of the expected enlarged ventricles and smaller exit route for CSF accompanying hydrocephalus, our *Sspo*^−/−^ mice had smaller ventricles at 6-weeks of age compared to wild type controls and no observable abnormalities in the cerebral aqueduct. Therefore, it seems unlikely that absence of RF alone is sufficient to cause aqueductal stenosis or hydrocephalus in mice. Data from humans are sparse. Although SCO damage is reported in 2 cases of human fetal hydrocephalus [35, 36], these observations were made after hydrocephalus had already developed, and therefore do not support causation. Unlike the human cases and rodent models mentioned above where severe SCO damage is observed, in our *Sspo−/−* mice, the SCO appeared normal by gross histology. Future studies implementing more in-depth characterization of the SCO developmental timeline may reveal subtle structural changes during SCO development. Because the *Sspo−/−* mice are germline knockout of *Sspo* and therefore lack the monomeric SSPO, we do not expect any formation of the RF polymer. Our genetic KO model therefore differs from the rat model, which was generated by antibody immunization against solubilized RF material [33, 34]. The guide RNAs we used to generate our mouse line targeted exon 2 and caused a 5 bp deletion and early stop codon in this exon. This deletion leads to a truncated SSPO protein missing the EMI domain critical for multimerization of EMI proteins [37]. Hypothetically, multimerization would be required for the aggregation of SSPO monomers into a fiber and fibers would not be seen in the absence of EMI [38]. This concept has been confirmed in zebrafish in which only 5 amino acids added to the EMI domain prevented the aggregation of SCO-spondin into a fiber [11]. The differences in SCO damage and absence of RF between our study compared to previous reports in rats may be explained by the different effects of genetic knockout versus immunization approaches.

Small ventricles in 6-week-old *Sspo*^−/−^ mice are consistent with reduced circulating CSF volume. However, the underlying mechanisms remain speculative. The earliest reported presence of soluble SSPO in mice is embryonic day 14 (E14) [39], so we would expect deficiency effects to manifest after this time. We did not measure ventricle sizes in embryonic mice (due to technical limitations of live MRI) or postnatal mice younger than 6 weeks. While live MRI of neonates is technically feasible [40–45], our laboratory’s protocols are not approved to perform live MRI until P14. Consequently, the exact developmental stage at which ventricle size in mutants first diverges from controls remains to be determined. CSF volume regulation is complex and remains poorly understood. However, it clearly depends on the rate of CSF secretion, fluid pressure, osmotic gradients, and flow patterns related to ciliary motion of CSF-contacting cells [46, 47]. These parameters all change as the brain matures [19, 48]. One remarkable property of SSPO is its ability to bind with numerous CSF proteins (reviewed in [2]). It is possible that SSPO and these numerous binding partners regulate CSF osmolarity. If so, osmolarity could mediate *Sspo*-deficiency effects on ventricle expansion in early development [49]. Specifically, by lacking SSPO protein, the developing ventricles might draw less water from nearby sources, resulting in lower CSF volume. SSPO-deficiency could also influence CSF secretion, as SCO and/or other paraventricular structures may contribute CSF production together with the ChP. Finally, the RF itself may influence CSF flow by extending through the aqueduct, 4th ventricle, and central canal. The abovementioned possibilities are purely speculative at this point.

*Sspo*-deficiency had a very small impact on thoracic curvature in mice. This finding contrasts sharply with zebrafish *sspo* knockouts, which consistently show a curled down embryonic phenotype [11]. This phenotype persists to juvenile/adult stages [23, 50, 51] and resembles defects observed in mutants with defective cilia [52–55] and/or signaling of the urotensin pathway [50, 55–57]. We suspect this difference may reflect greater reliance of four-limbed animals on the proprioceptive system conveying mechanosensory feedback from the periphery to maintaining proper spinal alignment throughout development [24, 58]. The corresponding critical contribution of dorsal root ganglia might explain why their spinal geometry would be less sensitive to perturbations affecting the interoceptive mechanosensory system constituted of the RF and the CSF-cNs [14, 59–62]. To characterize the mild skeletal phenotype of *Sspo*^*−/−*^ mice, we designed a new pipeline for the quantitative analysis of spinal shape using rodent micro-CT data. Existing methods could quantify strong deformations in various mutant mouse strains [24, 25] but are not suited to detect subtle changes or thoroughly examine spinal shape. Previous solutions were usually a transposition of traditional clinical methodologies, involving the measurement of Cobb angles (the angle between the end vertebrae of an identified curved segment) using manual or semi-automated annotations of X-ray images [63]. Efforts to improve the measurement of spinal shape in rodents have remained limited to the processing of two-dimensional X-ray data [64] and therefore did not address artefactual effects due to variable animal placement (see Fig. 3A). Possible directions for estimating three-dimensional spinal geometry in rodents include the use of generalizable annotation tools adapted to micro-CT data such as DicomAnnotator [65] or the development of 3D reconstruction methods similar to the ones used by clinicians [66–68]. We opted for a custom annotation-based solution tailored to our specific need, i.e., estimating three-dimensional spinal trajectories without reconstructing individual vertebrae. One key element of our strategy is the calculation of a side projection image obtained from a stripe of voxels centered on the spinal midline, which greatly facilitates the visualization of vertebral landmarks such as the spinal canal and intervertebral spaces. Our method represents a tradeoff between simplicity of use (high), rapidity of development (high), complexity of the measure (low) and accuracy of the measurement (high). Our code is openly available (https://github.com/teamnbc/MouSpine) and will be adaptable to other studies with some adjustments to work with multiple data formats and non-prone positions. The companion code for performing the necessary corrections, aligning individual trajectories, computing average trajectories and calculating curvature angles is also available (https://github.com/teamnbc/MouSpineR). Future application of our approach may answer additional questions such as how spine curvature changes in *Sspo*^*−/−*^ mice during embryonic development or during aging.

Consistent with the mild anatomical phenotype localized to the thoracic region of the spine, behaviors tested in *Sspo*^−/−^ mice were largely unaffected. Forelimbs connect to the thoracic region, guiding our choice of gripping strength and other motor behaviors. In gripping strength and other motor behaviors tested (open field, rotarod, and Digigait tests), *Sspo*^−/−^ mice did not show behavioral differences compared to their wild type littermate controls. These data agree with prior work in zebrafish, where muscle development was not affected by mutated SCO-spondin [23]. Our data do not rule out the existence of mild phenotypes that could be uncovered by an extensive suite of fine motor tests as have been applied to mice following ablation of CSF-cNs [60, 61]. Overall, the defects associated with loss of the RF are less pronounced in mice compared to deficits observed in zebrafish [59].

A limitation of our study is that it does not illuminate long term effects of SSPO loss. Regarding ventricle size, there is a dearth of clinical data from which to speculate. We could find only one observational study [69] which reported a correlation between smaller lateral ventricles and clinical outcome in higher levels of activity, anger, and irritability in six-week-old infants. The data in that study do not speak to cause-and-effect or long-term outcomes. Surgical shunts placed in hydrocephalus patients can cause small ventricles by over-drainage of CSF. This condition known as “slit ventricle syndrome” typically presents with debilitating chronic headache [70]. Despite much conjecture regarding the cause of slit ventricle syndrome [70], no consensus has been reached. Nonetheless, the differences between small ventricles produced by surgical interventions vs. small ventricles produced by developmental processes preclude informing predictions about long-term effects. Clearly, more study is needed, but in the absence of evidence to the contrary, we would not predict profound consequences of smaller ventricles per se based on our findings. Rather, our data underscore the relative adaptability of the ventricles to sustain moderate volume changes without overt disruption of brain functions. On the other hand, we can speculate that reduced CSF volume could have a cumulative lifelong impact on CSF clearance of toxins, transport and distribution of nutritive and growth factors, the motility of CSF-facing cilia which is tightly related to CSF flow, and adult neurogenesis [71, 72].

## Conclusions

In conclusion, the phenotype of our new genetic mouse *Sspo*^−/−^ model suggests that SSPO and RF are not crucial for spine morphogenesis in rodents as they are in zebrafish. Rather, it is likely that SSPO is one of the many factors that contribute to early life CSF formation and dynamics.

### Supplementary Information

**Additional file 1. **Statistical analysis of spine morphology.

**Additional file 2.** Additional data from digigait and open field tests.

**Additional file 3.** Supplemental figures and legends.

## Data Availability

Novel reagents are available from the corresponding author or a designated repository. The datasets used and/or analyzed during the current study are available from the corresponding author on reasonable request and made available on Dryad: All code is available on github: https://github.com/teamnbc/MouSpine https://github.com/teamnbc/MouSpineR

## Abbreviations

CSF: Cerebrospinal fluid
CSF-cN: Cerebrospinal fluid contacting neurons
RF: Reissner’s fiber
SCO: Subcommissural organ
SSPO: SCO-spondin
