# Supplementary material for: SCO-spondin knockout mice exhibit small brain ventricles and mild spine deformation
Source: Fluids Barriers CNS. 2023 Dec 5;20:89. doi: 10.1186/s12987-023-00491-8 (PMC10696872; doi:10.1186/s12987-023-00491-8)
Supplement: Supplementary file 1 — Additional file 1. Statistical analysis of spine morphology. [file 12987_2023_491_MOESM1_ESM.pdf]

Statistical analysis

FX Lejeune - Data and Analysis Core

2023-06-22

Contents

1 Method **2**

2 Data **3**

3 Plot **3**

4 LMM 6 weeks **4**

4.1 Type II Wald Chi-square tests . . . 4

4.2 Posthoc emmeans comparisons . . . 4

4.3 QC . . . 5

5 LMM 12 weeks **6**

5.1 Type II Wald Chi-square tests . . . 6

5.2 Posthoc emmeans comparisons . . . 6

5.3 QC . . . 7

6 R session information **8**

Statistical tests for Guillaume Dugué and Claire Wyart

1 Method

All statistical analyses were conducted using R version 4.2.2 (R Development Core Team, 2022) and plots were generated with the ggplot2 package (v3.4.2) (Wickham, 2016).

Differences in the angle of curvature were investigated for the two genotypes across the regions of the spine. Group differences were examined using a linear mixed-effects models (LMMs) fitted to the angle values. In the fitted models, the factors Genotype (-/-, +/+), Region (S1-S4, L1-L6, T1-T13) and Sex (M, F), and their interaction terms were regarded as fixed effects. The mice identifier was assigned as a random (intercept) effect to account for the paired measurements by animal across the spine locations. Two separate LMMs were fitted: one for the 6 weeks values and one for the 12 weeks values using restricted maximum-likelihood estimation (REML) with the function lmer in the lme4 package (Bates et al., 2015) (v1.1-31). For each model, significance of the main effects and interaction terms between Genotype, Region and Sex was assessed based on Type II Wald chi-square tests using the function Anova in the car package (v3.1-1), followed by posthoc comparisons of the two genotypes at each region of the spine within each sex using the emmeans package (v1.4.5) with False Discovery Rate (FDR) correction of P-values.

The level of statistical significance was set at p or adjusted $p < 0.05$ for all tests.

References

R Core Team (2022). R: A language and environment for statistical computing. R Foundation for Statistical Computing, Vienna, Austria. URL https://www.R-project.org/.

Wickham H. 2016. Ggplot2: Elegant Graphics for Data Analysis. Springer.

Bates D, Maechler M, Bolker B, Walker S. Fitting Linear Mixed Effects Models Using lme4. J Stat Softw. 2015;67:1–48.

2 Data

Number of missing values. Withdraw "Ca5", "Ca4", "Ca3", "Ca2", "Ca1", "Ce2" and "Ce1" as they contain a high number of missing values.

	Ca5	Ca4	Ca3	Ca2	Ca1	S4	Ce3	Ce2	Ce1
F__6w	23	19	3	0	0	0	0	24	24
M__6w	22	21	14	6	0	0	0	22	22
F__12w	22	22	18	9	1	0	1	22	22
M__12w	23	23	23	23	13	5	0	23	23

3 Plot

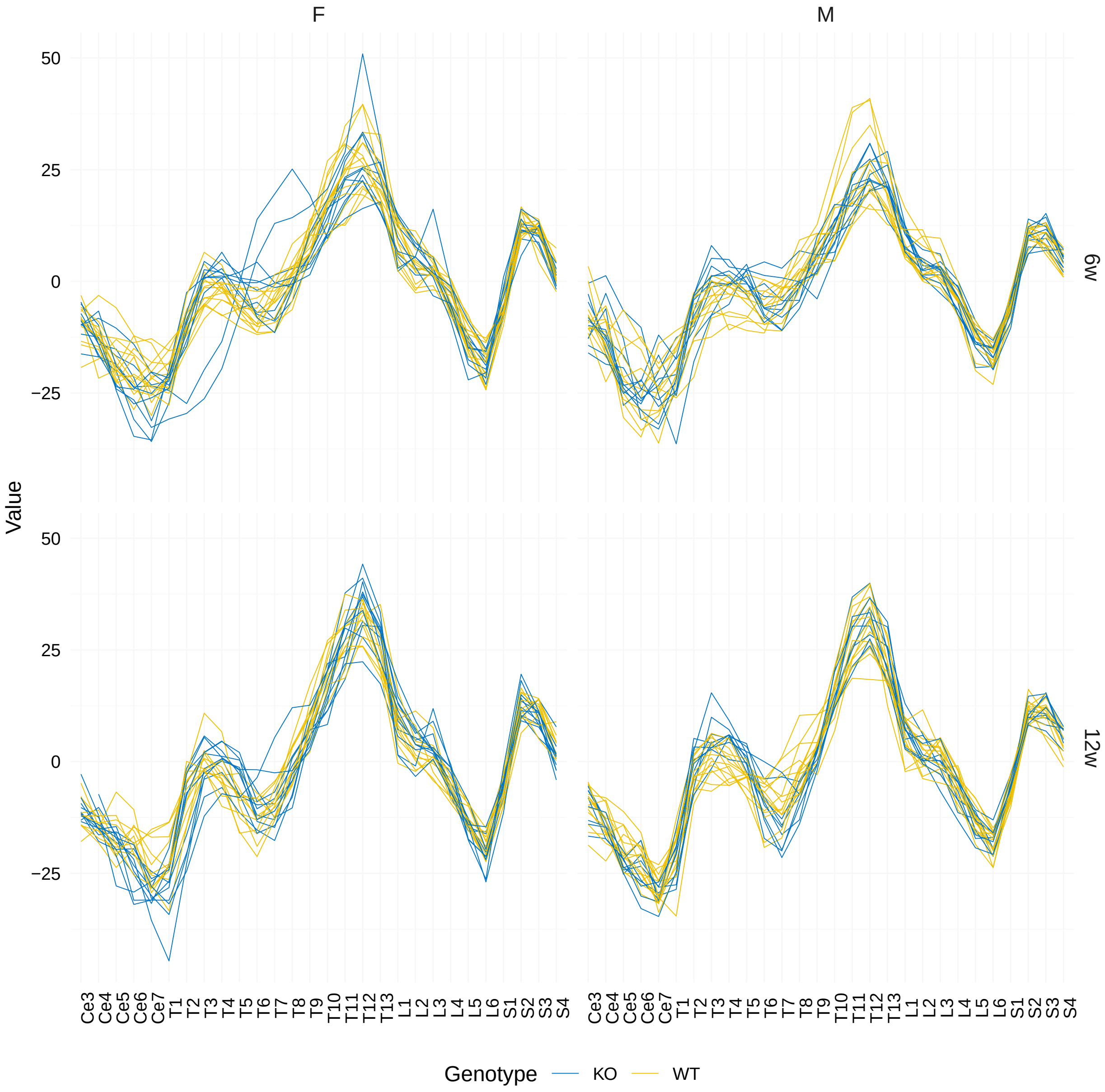

4 LMM 6 weeks

4.1 Type II Wald Chi-square tests

Type II Wald chi-square test revealed a non-significant trend towards an interaction effect of Genotype, Region and Sex on the angle of curvature ($\chi^2(27) = 39.0$, $p = 0.063$).

```
## Analysis of Deviance Table (Type II Wald chisquare tests)
##
## Response: Value
##                          Chisq Df Pr(>Chisq)
## Genotype                0.2011  1  0.6538527
## Region              10669.0495 27  < 2.2e-16 ***
## Sex                     2.1869  1  0.1391922
## Genotype:Region        57.0855 27  0.0006273 ***
## Genotype:Sex            0.6042  1  0.4369771
## Region:Sex             42.3933 27  0.0300909 *
## Genotype:Region:Sex    39.0240 27  0.0630417 .
## ---
## Signif. codes:  0 '***' 0.001 '**' 0.01 '*' 0.05 '.' 0.1 ' ' 1
```

4.2 Posthoc emmeans comparisons

4.2.1 Female - 6 weeks

Difference at T6 ($p = 0.004$).

Table 2: Female - 6 weeks

contrast	Region	estimate	SE	df	t.ratio	p.value	padj
KO - WT	T1	-3.100	1.932	1176	-1.604	0.10894	0.41760
KO - WT	T2	-4.099	1.932	1176	-2.121	0.03410	0.22580
KO - WT	T3	-1.769	1.932	1176	-0.916	0.36002	0.72471
KO - WT	T4	1.004	1.932	1176	0.520	0.60333	0.85877
KO - WT	T5	3.755	1.932	1176	1.943	0.05223	0.24026
KO - WT	T6	7.288	1.932	1176	3.772	0.00017	0.00391
KO - WT	T7	4.909	1.932	1176	2.541	0.01119	0.12868
KO - WT	T8	2.346	1.932	1176	1.214	0.22501	0.62156
KO - WT	T9	-0.887	1.932	1176	-0.459	0.64624	0.85877
KO - WT	T10	-3.988	1.932	1176	-2.064	0.03927	0.22580
KO - WT	T11	-1.704	1.932	1176	-0.882	0.37811	0.72471
KO - WT	T12	0.339	1.932	1176	0.176	0.86058	0.91607
KO - WT	T13	-0.591	1.932	1176	-0.306	0.75961	0.91607
KO - WT	L1	-0.318	1.932	1176	-0.165	0.86925	0.91607
KO - WT	L2	2.588	1.932	1176	1.339	0.18068	0.59366
KO - WT	L3	1.320	1.932	1176	0.683	0.49471	0.85877
KO - WT	L4	-0.846	1.932	1176	-0.438	0.66161	0.85877
KO - WT	L5	-2.256	1.932	1176	-1.168	0.24322	0.62156
KO - WT	L6	0.093	1.932	1176	0.048	0.96180	0.96180
KO - WT	S1	1.840	1.932	1176	0.952	0.34105	0.72471
KO - WT	S2	-0.818	1.932	1176	-0.423	0.67208	0.85877
KO - WT	S3	-0.301	1.932	1176	-0.156	0.87624	0.91607
KO - WT	S4	-0.826	1.932	1176	-0.427	0.66916	0.85877

4.2.2 Male - 6 weeks

No difference.

Table 3: Male - 6 weeks

contrast	Region	estimate	SE	df	t.ratio	p.value	padj
KO - WT	T1	-3.661	1.998	1176	-1.832	0.06720	0.38640
KO - WT	T2	0.680	1.998	1176	0.340	0.73367	0.95965
KO - WT	T3	3.925	1.998	1176	1.964	0.04975	0.38142
KO - WT	T4	4.241	1.998	1176	2.122	0.03403	0.38142
KO - WT	T5	5.331	1.998	1176	2.668	0.00774	0.17802
KO - WT	T6	1.648	1.998	1176	0.825	0.40975	0.80487
KO - WT	T7	-1.396	1.998	1176	-0.698	0.48502	0.80487
KO - WT	T8	-3.253	1.998	1176	-1.628	0.10384	0.47766
KO - WT	T9	-2.555	1.998	1176	-1.279	0.20125	0.51431
KO - WT	T10	-2.822	1.998	1176	-1.412	0.15821	0.49269
KO - WT	T11	-2.980	1.998	1176	-1.491	0.13613	0.49269
KO - WT	T12	-1.248	1.998	1176	-0.624	0.53253	0.80487
KO - WT	T13	2.735	1.998	1176	1.369	0.17137	0.49269
KO - WT	L1	1.674	1.998	1176	0.838	0.40239	0.80487
KO - WT	L2	-1.263	1.998	1176	-0.632	0.52760	0.80487
KO - WT	L3	-0.031	1.998	1176	-0.015	0.98777	0.98777
KO - WT	L4	-0.053	1.998	1176	-0.027	0.97879	0.98777
KO - WT	L5	-0.394	1.998	1176	-0.197	0.84387	0.95965
KO - WT	L6	0.311	1.998	1176	0.156	0.87620	0.95965
KO - WT	S1	-0.456	1.998	1176	-0.228	0.81935	0.95965
KO - WT	S2	-1.176	1.998	1176	-0.589	0.55629	0.80487
KO - WT	S3	1.165	1.998	1176	0.583	0.55991	0.80487
KO - WT	S4	0.492	1.998	1176	0.246	0.80561	0.95965

4.3 QC

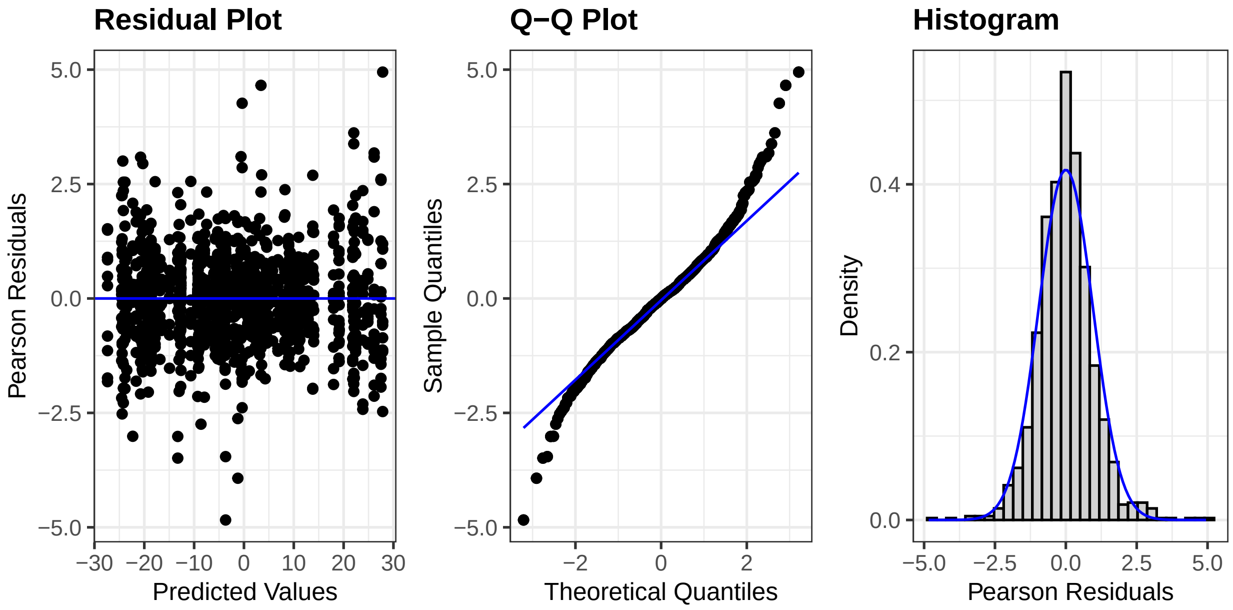

5 LMM 12 weeks

5.1 Type II Wald Chi-square tests

Type II Wald chi-square test indicated a significant interaction effect of Genotype, Region and Sex on the angle of curvature ($\chi^2(27) = 52.7$, $p = 0.002$).

```
## Analysis of Deviance Table (Type II Wald chisquare tests)
##
## Response: Value
##                           Chisq Df Pr(>Chisq)
## Genotype                 1.9832  1   0.159053
## Region               16573.1750 27  < 2.2e-16 ***
## Sex                      0.9580  1   0.327684
## Genotype:Region        110.6582 27  4.296e-12 ***
## Genotype:Sex             0.8869  1   0.346306
## Region:Sex             166.2530 27  < 2.2e-16 ***
## Genotype:Region:Sex     52.7037 27   0.002185 **
## ---
## Signif. codes:  0 '***' 0.001 '**' 0.01 '*' 0.05 '.' 0.1 ' ' 1
```

5.2 Posthoc emmeans comparisons

5.2.1 Female - 12 weeks

Group differences at T1 ($p = 0.003$), T2 ($p = 0.043$) and T5 ($p = 0.007$).

Table 4: Female - 12 weeks

contrast	Region	estimate	SE	df	t.ratio	p.value	padj
KO - WT	T1	-6.719	1.757	1142	-3.825	0.00014	0.00322
KO - WT	T2	-4.873	1.757	1142	-2.774	0.00563	0.04316
KO - WT	T3	-1.751	1.757	1142	-0.997	0.31912	0.46578
KO - WT	T4	3.195	1.757	1142	1.819	0.06917	0.26515
KO - WT	T5	6.049	1.757	1142	3.443	0.00060	0.00690
KO - WT	T6	2.872	1.757	1142	1.635	0.10238	0.29434
KO - WT	T7	-0.397	1.757	1142	-0.226	0.82125	0.84939
KO - WT	T8	-1.855	1.757	1142	-1.056	0.29135	0.46578
KO - WT	T9	-2.292	1.757	1142	-1.305	0.19225	0.40198
KO - WT	T10	-4.224	1.757	1142	-2.405	0.01634	0.09395
KO - WT	T11	-0.875	1.757	1142	-0.498	0.61865	0.71145
KO - WT	T12	3.049	1.757	1142	1.736	0.08288	0.27232
KO - WT	T13	2.752	1.757	1142	1.567	0.11746	0.30018
KO - WT	L1	1.489	1.757	1142	0.848	0.39684	0.48039
KO - WT	L2	1.490	1.757	1142	0.848	0.39643	0.48039
KO - WT	L3	3.574	1.757	1142	2.034	0.04215	0.19389
KO - WT	L4	1.925	1.757	1142	1.096	0.27331	0.46578
KO - WT	L5	-2.070	1.757	1142	-1.178	0.23889	0.45787
KO - WT	L6	-1.501	1.757	1142	-0.854	0.39316	0.48039
KO - WT	S1	0.334	1.757	1142	0.190	0.84939	0.84939
KO - WT	S2	0.655	1.757	1142	0.373	0.70949	0.77706
KO - WT	S3	-1.733	1.757	1142	-0.987	0.32402	0.46578
KO - WT	S4	-2.567	1.757	1142	-1.461	0.14417	0.33159

5.2.2 Male - 12 weeks

Differences at T4 ($p = 0.005$), T5 ($p = 0.048$), T7 ($p = 0.014$) and T8 ($p = 0.005$).

Table 5: Male - 12 weeks

contrast	Region	estimate	SE	df	t.ratio	p.value	padj
KO - WT	T1	-1.693	1.760	1142	-0.962	0.33644	0.64484
KO - WT	T2	2.059	1.760	1142	1.170	0.24237	0.58949
KO - WT	T3	4.244	1.760	1142	2.411	0.01605	0.07383
KO - WT	T4	6.204	1.760	1142	3.524	0.00044	0.00506
KO - WT	T5	4.654	1.760	1142	2.644	0.00831	0.04778
KO - WT	T6	-0.082	1.760	1142	-0.047	0.96275	0.99196
KO - WT	T7	-5.513	1.760	1142	-3.132	0.00178	0.01365
KO - WT	T8	-6.386	1.760	1142	-3.628	0.00030	0.00506
KO - WT	T9	-2.920	1.760	1142	-1.659	0.09739	0.32000
KO - WT	T10	0.625	1.760	1142	0.355	0.72274	0.87490
KO - WT	T11	0.483	1.760	1142	0.274	0.78389	0.90147
KO - WT	T12	1.044	1.760	1142	0.593	0.55340	0.74872
KO - WT	T13	3.284	1.760	1142	1.866	0.06236	0.23905
KO - WT	L1	1.554	1.760	1142	0.883	0.37753	0.66794
KO - WT	L2	0.018	1.760	1142	0.010	0.99196	0.99196
KO - WT	L3	-1.999	1.760	1142	-1.136	0.25630	0.58949
KO - WT	L4	-2.174	1.760	1142	-1.235	0.21701	0.58949
KO - WT	L5	-1.275	1.760	1142	-0.724	0.46917	0.71939
KO - WT	L6	0.326	1.760	1142	0.185	0.85308	0.93433
KO - WT	S1	1.383	1.760	1142	0.786	0.43216	0.70998
KO - WT	S2	-1.071	1.760	1142	-0.608	0.54308	0.74872
KO - WT	S3	0.841	1.760	1142	0.478	0.63301	0.80885
KO - WT	S4	2.090	1.943	1142	1.076	0.28214	0.58993

5.3 QC

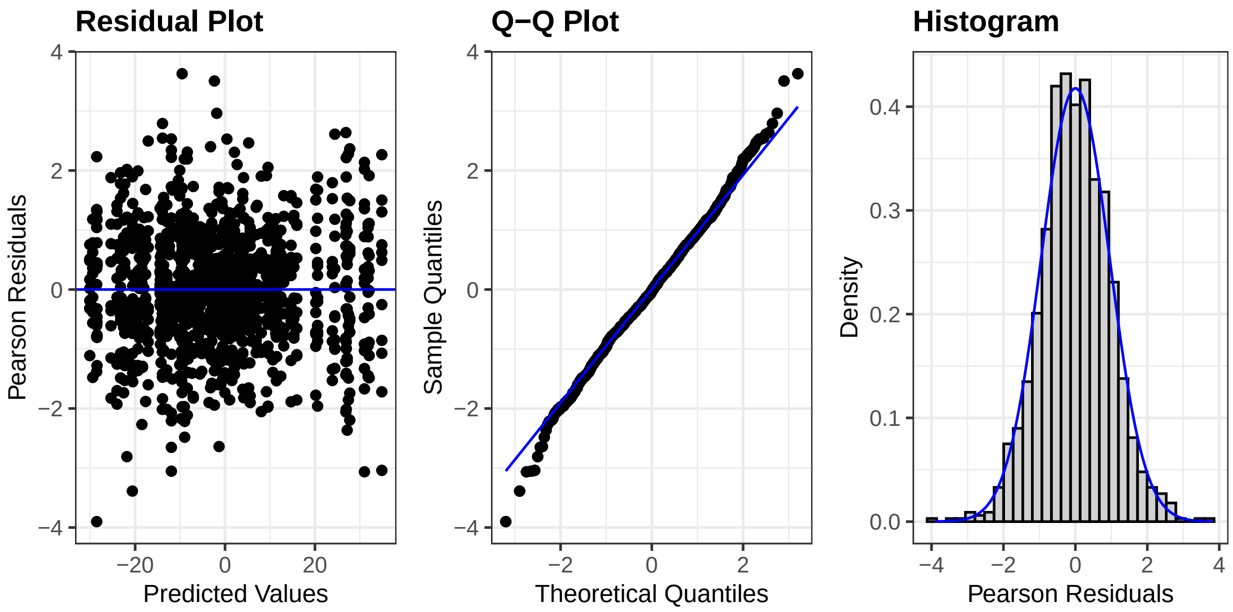

6 R session information

```
## R version 4.2.2 (2022-10-31 ucrt)
## Platform: x86_64-w64-mingw32/x64 (64-bit)
## Running under: Windows 10 x64 (build 17134)
##
## Matrix products: default
##
## locale:
## [1] LC_COLLATE=French_France.1252  LC_CTYPE=French_France.1252
## [3] LC_MONETARY=French_France.1252 LC_NUMERIC=C
## [5] LC_TIME=French_France.1252
##
## attached base packages:
## [1] stats     graphics  grDevices utils     datasets  methods   base
##
## other attached packages:
##  [1] knitr_1.42         factoextra_1.0.7   ggResidpanel_0.3.0 emmeans_1.8.2
##  [5] car_3.1-1          carData_3.0-5      lme4_1.1-31        Matrix_1.5-3
##  [9] reshape2_1.4.4     plyr_1.8.8         ggsci_2.9          ggplot2_3.4.2
##
## loaded via a namespace (and not attached):
##  [1] httr_1.4.5              tidyr_1.2.1             jsonlite_1.8.4
##  [4] viridisLite_0.4.1       splines_4.2.2           foreach_1.5.2
##  [7] qqconf_1.3.1            memuse_4.2-3            ggrepel_0.9.2
## [10] robustbase_0.95-1       yaml_2.3.6              backports_1.4.1
## [13] pillar_1.9.0            lattice_0.20-45         glue_1.6.2
## [16] digest_0.6.30           minqa_1.2.5             colorspace_2.0-3
## [19] sandwich_3.0-2          cowplot_1.1.1           htmltools_0.5.5
## [22] pkgconfig_2.0.3         broom_1.0.4             twosamples_2.0.0
## [25] purrr_0.3.5             xtable_1.8-4            mvtnorm_1.1-3
## [28] scales_1.2.1            pracma_2.4.2            tibble_3.2.1
## [31] farver_2.1.1            generics_0.1.3          TH.data_1.1-1
## [34] withr_2.5.0             opdisDownsampling_0.8.2 lazyeval_0.2.2
## [37] pbkrtest_0.5.2          cli_3.4.1               survival_3.4-0
## [40] magrittr_2.0.3          estimability_1.4.1      evaluate_0.20
## [43] fansi_1.0.3             doParallel_1.0.17       nlme_3.1-160
## [46] MASS_7.3-58.1           benchmarkme_1.0.8       tools_4.2.2
## [49] data.table_1.14.4       lifecycle_1.0.3         multcomp_1.4-23
## [52] stringr_1.5.0           plotly_4.10.1           munsell_0.5.0
## [55] qqplotr_0.0.6           compiler_4.2.2          caTools_1.18.2
## [58] rlang_1.1.0             grid_4.2.2              nloptr_2.0.3
## [61] iterators_1.0.14        rstudioapi_0.14         htmlwidgets_1.6.2
## [64] labeling_0.4.2          bitops_1.0-7            rmarkdown_2.21
## [67] boot_1.3-28.1           gtable_0.3.3            codetools_0.2-19
## [70] abind_1.4-5             DBI_1.1.3               benchmarkmeData_1.0.4
## [73] R6_2.5.1                zoo_1.8-11              dplyr_1.0.10
## [76] fastmap_1.1.0           utf8_1.2.2              stringi_1.7.8
## [79] parallel_4.2.2          Rcpp_1.0.9              vctrs_0.6.1
## [82] DEoptimR_1.0-11         tidyselect_1.2.0        xfun_0.38
## [85] coda_0.19-4
```
