# Supplementary material for: SCO-spondin knockout mice exhibit small brain ventricles and mild spine deformation
Source: Fluids Barriers CNS. 2023 Dec 5;20:89. doi: 10.1186/s12987-023-00491-8 (PMC10696872; doi:10.1186/s12987-023-00491-8)
Supplement: Supplementary file 3 — Additional file 3. Supplemental figures and legends. [file 12987_2023_491_MOESM3_ESM.docx]

*Figure S1*

*
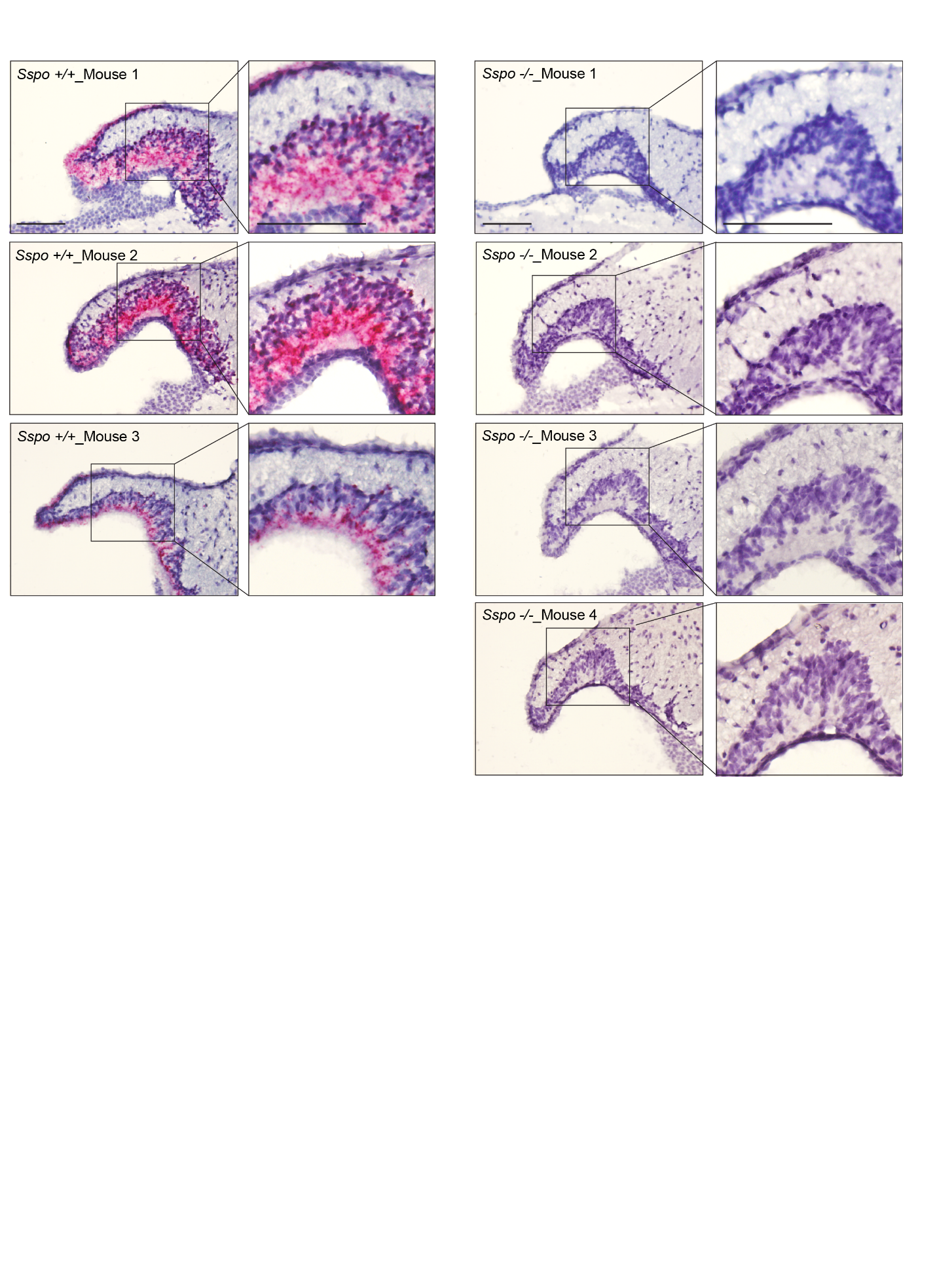
*

**Figure S1.** Histological images with hematoxylin staining showing no noticeable deficits in the SCO structure and nuclei arrangement of *Sspo^-/-^* mice compared to *Sspo^+/+^* mice. Tissues were collected from 12 weeks old mice. Sagittal sections were used. Scale = 100 µm.

*Figure S2*


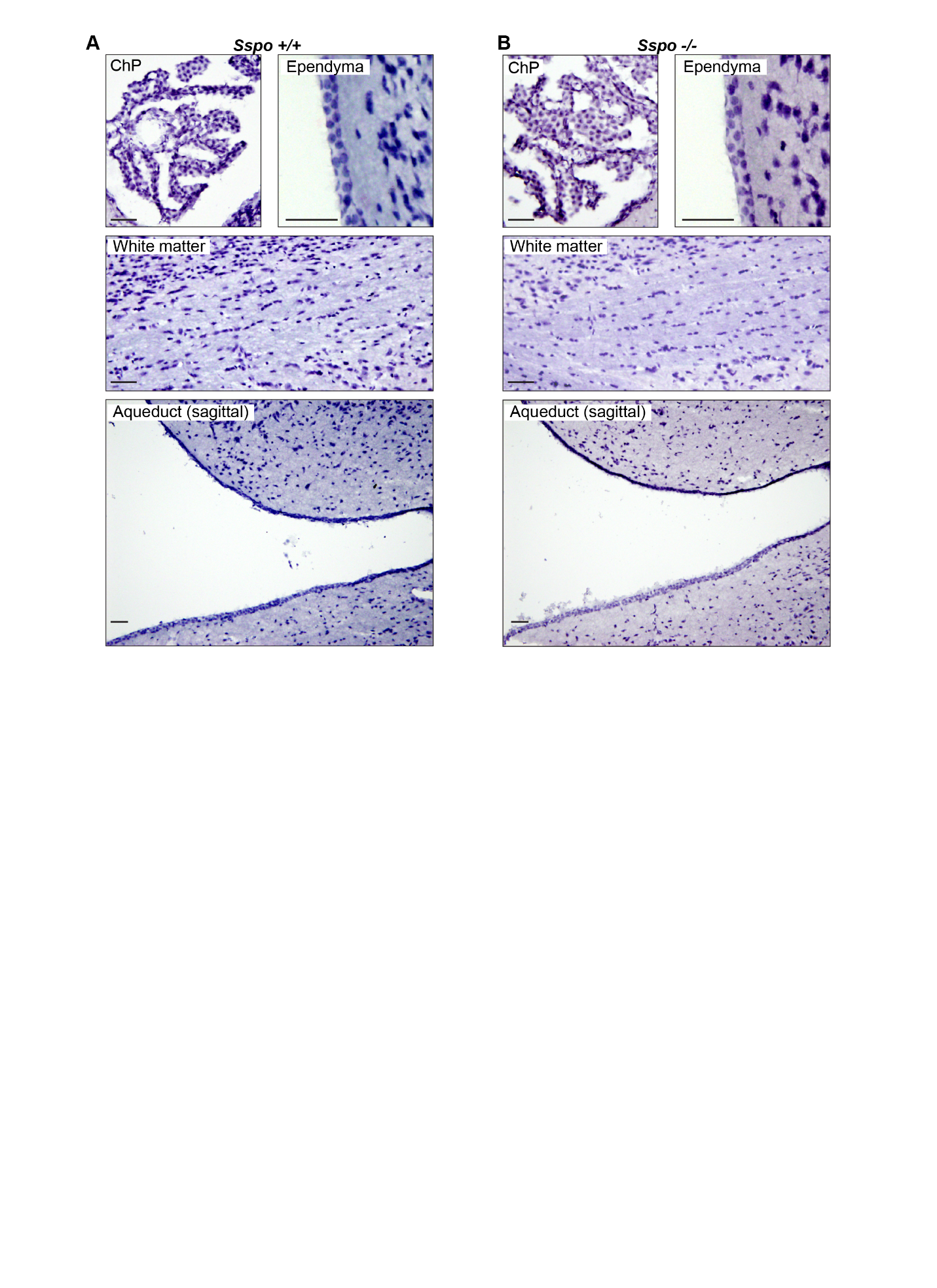


**Figure S2.** Histological images with hematoxylin staining showing no noticeable deficits in the choroid plexus, ependyma, white matter, and aqueduct of *Sspo^-/-^* mice (B) compared to *Sspo^+/+^* mice (A). Tissues were collected from 12 weeks old mice. Sagittal sections were used. Scale = 50 µm.

*Figure S3*


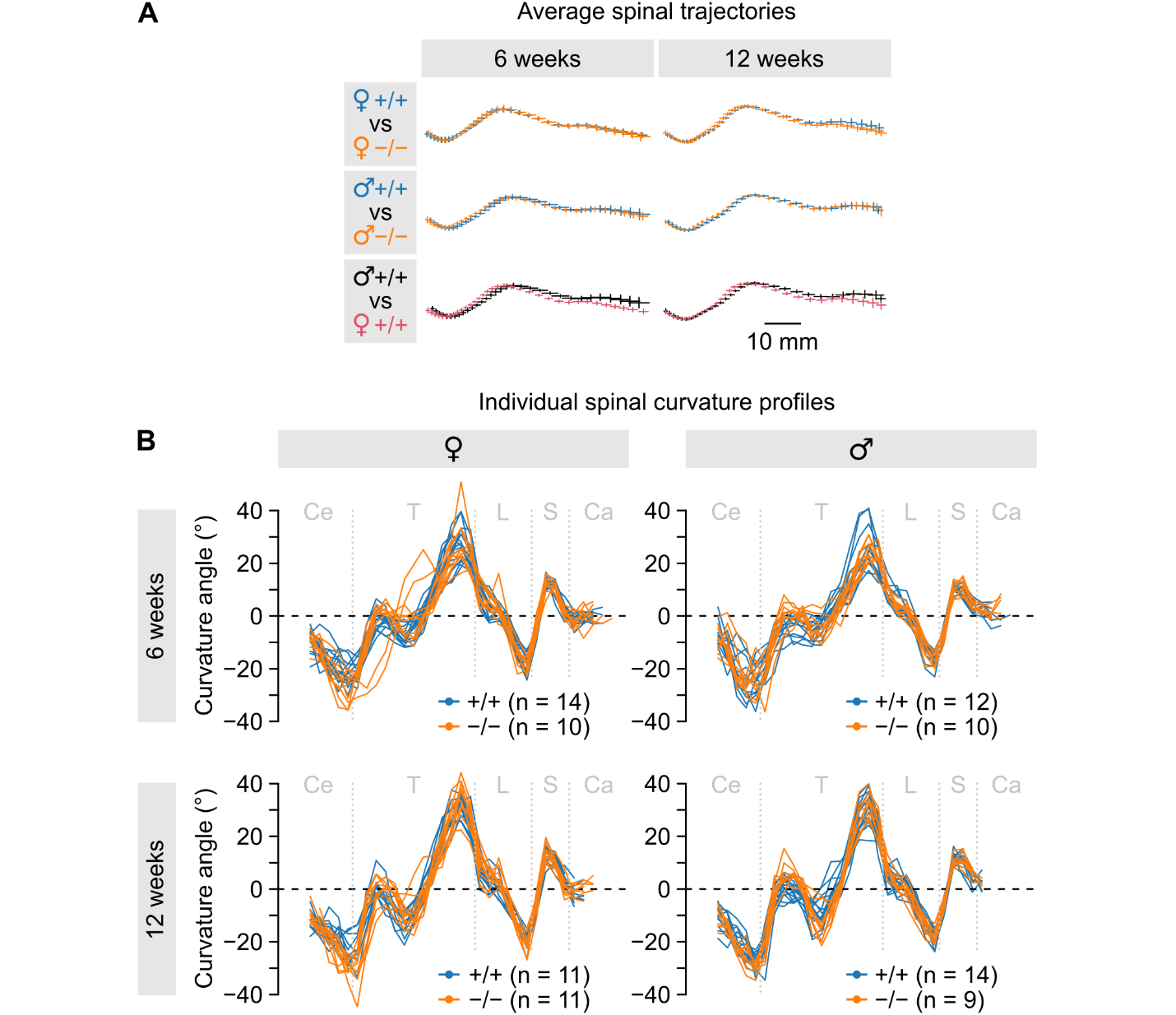


**Figure S3.** **Additional information on the analysis of micro-CT data**. (**A**) Optimal alignment of average spinal trajectories computed for different mouse groups. For each trajectory, the mean (*x*, *z*) position of reference points is marked using a cross whose horizontal and vertical bars represent the S.D. along *x* and *z*, respectively. (**B**) Superimposed individual curvature profiles for all mouse groups.
